# Challenge accepted: uncovering the role of rare genetic variants in Alzheimer’s disease

**DOI:** 10.1186/s13024-021-00505-9

**Published:** 2022-01-09

**Authors:** Marzieh Khani, Elizabeth Gibbons, Jose Bras, Rita Guerreiro

**Affiliations:** 1 School of Biology, College of Science, University of Tehran, Tehran, Iran; 2 Department of Neurodegenerative Science, Van Andel Institute, 333 Bostwick Ave. N.E., Grand Rapids, Michigan 49503-2518 USA; 3 Division of Psychiatry and Behavioral Medicine, Michigan State University College of Human Medicine, Grand Rapids, MI USA

**Keywords:** Alzheimer’s disease, Genetic architecture, Early-onset AD, Late-onset AD, Rare variants, Genetics

## Abstract

The search for rare variants in Alzheimer’s disease (AD) is usually deemed a high-risk - high-reward situation. The challenges associated with this endeavor are real. Still, the application of genome-wide technologies to large numbers of cases and controls or to small, well-characterized families has started to be fruitful.

Rare variants associated with AD have been shown to increase risk or cause disease, but also to protect against the development of AD. All of these can potentially be targeted for the development of new drugs.

Multiple independent studies have now shown associations of rare variants in *NOTCH3*, *TREM2*, *SORL1*, *ABCA7*, *BIN1*, *CLU*, *NCK2*, *AKAP9*, *UNC5C*, *PLCG2,* and *ABI3* with AD and suggested that they may influence disease via multiple mechanisms. These genes have reported functions in the immune system, lipid metabolism, synaptic plasticity, and apoptosis. However, the main pathway emerging from the collective of genes harboring rare variants associated with AD is the Aβ pathway. Associations of rare variants in dozens of other genes have also been proposed, but have not yet been replicated in independent studies. Replication of this type of findings is one of the challenges associated with studying rare variants in complex diseases, such as AD. In this review, we discuss some of these primary challenges as well as possible solutions.

Integrative approaches, the availability of large datasets and databases, and the development of new analytical methodologies will continue to produce new genes harboring rare variability impacting AD. In the future, more extensive and more diverse genetic studies, as well as studies of deeply characterized families, will enhance our understanding of disease pathogenesis and put us on the correct path for the development of successful drugs.

**Supplementary Information:**

The online version contains supplementary material available at 10.1186/s13024-021-00505-9.

## Background

The main goals of human genetics include the improvement of diagnosis and prognosis of human disease, as well as the identification of therapeutic targets. To accomplish this, it is crucial to fully understand the genetic architecture of diseases, more specifically, to have complete knowledge of all genetic contributions to a given disease outcome and the characteristics of those contributions [1].

Alzheimer’s disease (AD; Online Mendelian Inheritance in Man [OMIM]#104300) has a complex nature with contributions from multiple environmental and genetic factors. Clinically is characterized by deficits in short-term memory formation and additional cognitive functions such as word-finding, spatial cognition, reasoning, judgment, and problem-solving [2, 3]. Neuropathologically, it is associated with hallmarks such as the presence of extracellular depositions of amyloid-beta (Aβ) peptides and intracellular neurofibrillary tangles. The former are a byproduct of sequential cleavage of the amyloid precursor protein (APP) by the enzymatic complexes beta (β) and gamma (γ) secretase, and the latter are composed of hyperphosphorylated tau protein [4, 5]. Genetically, AD is also complex. According to the age at onset of clinical symptoms, it can be divided into two categories: early-onset AD (EOAD), if symptoms occur before 65 years, and late-onset AD (LOAD), if symptoms occur after that age. Both forms of the disease can be subdivided into familial and sporadic, according to the presence or absence of a family history of the disease, respectively. Both early and late-onset forms of AD are considered to have a high degree of heritability, ranging from 90-100% in EOAD to 60-80% in LOAD [6, 7]. Most sporadic cases present with late-onset symptoms, and familial inheritance of AD is typically associated with early-onset forms of the disease. Some AD cases are monogenic, where mutations in one gene cause the disease; the vast majority are sporadic with a polygenic basis. Both common and rare variants contribute to risk and phenotype. Even if considered distinct forms of AD, EOAD and LOAD overlap significantly in clinical, pathological, and genetic features. This means that understanding the genetic architecture of both forms of the disease will be essential to identify potential therapeutic targets, an elusive but critical task given the current absence of effective disease-modifying drugs.

Over the years, considerable advances have been made in characterizing the genes and genetic variants involved in this disease. These advances have been tightly related to developments in genetic technologies, such as the development of platforms for genome-wide association studies and sequencing of exomes and genomes. Still, the genetic factors identified so far account only for a portion of the underlying genetic basis of disease. Thus, we are far from having a complete understanding of the genetic architecture of AD.

With improvements of the technologies and their application to ever-growing numbers of samples, we expect other genes with a role in disease to be identified. Notably, we have most recently seen an increase in the number of rare variants associated with AD, despite the significant challenges related to studying this type of variant. In this review, we will focus on the contributions of rare variants to AD in the context of its overall genetic architecture.

## The genetic architecture of Alzheimer’s disease

The genetic architecture of a disease can be defined as the set of variants influencing a phenotype, the magnitude of their respective effects on the phenotype, their population frequency, and their interactions with each other and the environment [1]. The complete knowledge of the genetic architecture of disease functions as a blueprint that can be used for translational efforts and drug development. The more detailed the blueprint, the more targets will be available for drug development and the better we will understand how they interact, leading to disease. The development of aducanumab is a prime example of this for AD. This is an amyloid beta-directed monoclonal antibody, recently approved by the US Food and Drug Administration, that was generally based on the initial genetic findings of *APP*, *PSEN1*, and *PSEN2* mutations as causes of AD and the subsequent development of the amyloid cascade hypothesis [8].

### Types of variants and approaches to study them

Variants in the human genome can range from single nucleotide variants (SNVs) to large structural changes, including copy number variants, translocations, and inversions [9]. All of these can be common (when presenting a minor allele frequency, MAF ≥ 5%), have a low frequency (MAF ≥ 1% and < 5%), be rare (MAF ≥ 0.1% and < 1%), or be ultra-rare (MAF < 0.1%, many with the plurality observed only once) in the population. In order to understand the genetic architecture of disease, all the different types of genetic changes need to be assessed and understood in a biological context. Different genetic mapping techniques have been developed that allow the determination of associations between sequence variants and phenotypic variability. Some of these techniques are ideal for testing common variants, while others are more appropriate to assess rare variability. In the past decade, these methodologies have started to interrogate the genome leading to a much more comprehensive view of genetic variability associated with disease.

The first of these methodologies to be applied to the study of AD were genome-wide association studies (GWAS). These platforms use genome-wide genotyping arrays to measure genetic variation and test the association of AD with common genetic variants by typically comparing frequencies of variants between cases and controls. Different designs of genotyping arrays and the use of imputation strategies have, more recently, allowed for the assessment of lower frequency and rare variants in GWAS.

Direct sequencing is the primary technology for the detection of rare genetic variation. Next-generation sequencing (NGS) technologies in the form of exome and genome sequencing (ES and GS) and several additional cost-effective approaches including targeted sequencing of selected loci, directed genotyping, and improved imputation of large cohorts have been developed for this purpose [10, 11]. The use of exome-wide microarrays with variants selected from exome sequencing is an alternate approach for the detection of rare variants. The main limitation of genotyping approaches is that they can only test what is known [10]. Sequencing technologies allow for the discovery of novel variants. Variants of small statistical effects can show substantial biological changes of disease relevance. It is important to note that even NGS technologies currently in heavy use have limitations, but approaches are being put into place to help. Repeat expansions (e.g. *C9orf72*) are not detectable in PCR-positive library preps (which have long been standard), but are readily detectable (even in short-read Illumina data) in PCR-free library preparations. Long-read sequencing is also being more commonly applied to allow better detection of copy number variants.

### Common variability contributing to AD

Common SNVs in *APOE* (apolipoprotein E) are the most well-known and significant genetic risk modulators for AD. Three main isoforms are encoded by different alleles and vary at positions 112 and 158 of the protein, either carrying a cysteine or an arginine (Transcript ID: ENST00000252486.9, Protein sequence ID: NP_000032.1). These are referred to as *APOE* ε2, ε3 and ε4, with *APOE* ε3 being the most common among human populations, and ε4 and ε2 increasing and decreasing the risk for AD, respectively, in a dose-dependent manner [12, 13]. Non-Hispanic white individuals harboring one or two copies of *APOE* ε4 have an average increased risk of developing AD of 3- and 15-fold, respectively. *APOE* ε4 has also been shown to modify the age at onset in both EOAD and LOAD, and it has been reported to have different effects in different populations [14, 15].

It was only with the application of GWAS to AD that additional, replicated genetic loci were identified to be associated with disease risk. The first GWAS in AD, most probably due to the small sample size, only identified *APOE* as a risk locus. This confirmed that genetic variation at this locus is the most significant common genetic risk factor for LOAD, and that genetic variation at other loci, individually, confer only minor effects on disease risk [16]. Large-scale GWAS involving over 15,000 samples started to identify other regions of interest in the genome, some of which consistently replicated across studies [17, 18]. Currently, with increased statistical power gained by the use of very large numbers of samples, over 70 risk loci with genome-wide significance have been associated with AD [19, 20]. Many of these associations were identified in non-coding regions and were found to be enriched at regulatory sites, with only 2% of variants associated with LOAD being located within exons [21]. In many cases, the true cause of the association is yet to be defined but the aggregation of loci according to their biological effects has led to a better understanding of the biological pathways involved in AD etiology. These include some obvious pathways, such as the Aβ and tau pathways, immune system and neuroinflammation pathways, and several others like lipid and glucose metabolism, synaptic plasticity, neurogenesis, axon outgrowth, blood-brain barrier integrity. These pathways are not independent of each other and often work in a synergic way in the central nervous system [10].

Results from GWAS have also been used to determine polygenic risk scores (PRS). These scores allow the individualized risk prediction in which an individual's risk for AD is determined by the combinatorial effect of multiple variants acting together across the genome [22]. PRS will be essential for the identification and stratification of at-risk individuals who may benefit from early therapeutic interventions. To this end, an approach has been proposed that, in addition to the typical PRS using common variants, also accounts for the varying disease prevalence in different genotype and age groups when modeling the *APOE* and rare genetic variants risk [23]. This is an important approach since rare variants, and in particular, singletons can have a substantial contribution to the heritability of complex diseases [24].

### Rare variants in Alzheimer’s disease

Heritability estimates for AD suggest that approximately only 33% of the genetic variance of sporadic AD is accounted for by common variants, indicating that the genetic architecture of AD is more complex than that proposed by the “common disease, common variant” hypothesis that GWAS are designed to test [25]. This puts ultra-rare, rare, and low-frequency variation (referred here as ‘rare variants’ for simplicity) in the spotlight as potential contributors to AD’s ‘missing heritability’ [24, 26, 27]. As per definition, this type of genetic variability will not contribute to disease in many individuals, making it difficult to understand their importance in disease and why we should study them. In fact, rare variants usually have larger effect sizes on disease risk, especially when compared to the common variants identified by GWAS, because they typically have a more deleterious impact on protein function. Additionally, as mentioned above, the methods used to identify rare variants associated with disease typically point to a specific gene (and not to a genomic region), making it easier to predict the effects of the variants by using cell or animal models.

Consequently, studying these rare variants often leads to a significantly improved understanding of disease mechanisms and can better pinpoint specific therapeutic targets. This has been the case for the development of the “amyloid cascade hypothesis”. It suggests that amyloid deposition is the initial causative event in a cascade of symptoms resulting in neurodegeneration and cognitive decline [8], which was based on the initial discovery of *APP* mutations as the cause of familial early-onset AD, leading to a central dogma that has guided research in AD for many years. Similarly, the more recent finding of *TREM2* rare variants as risk factors for AD has put immune cell function and inflammatory pathways at the center stage of AD research [28].

#### Genes harboring rare variants that cause AD

Linkage studies in multi-generational EOAD families with autosomal dominant inheritance identified mutations in three genes as the cause of the disease: amyloid precursor protein (*APP*), presenilin 1 (*PSEN1*), and presenilin 2 (*PSEN2*) [29,30]. In *APP,* more than 50 highly penetrant mutations have been identified, mainly localized near the secretases' cleavage sites and in the domain encoding for the Abeta peptide. Also, *APP* triplications (both as small events or in trisomy 21) can cause AD as well. In general, these mutations cause an increase in the production of amyloid or the propensity of amyloid aggregation. Similarly, mutations in *PSEN1* and *PSEN2* also result in an increased production of more aggregation-prone and longer species of Abeta. This occurs because both proteins are part of the γ-secretase complex responsible for APP processing [31]. Mutations in *PSEN1* contribute to around 80% of the monogenic forms of AD, while mutations in *PSEN2* are much less common. The clinical effects of mutations in *PSEN2* are more variable than those observed for *PSEN1* and *APP*. For example, ages at onset for *PSEN*2 mutation carriers range from 40 to 85 years of age [32], and some *PSEN2* mutations seem to exhibit reduced penetrance [33, 34].

Interestingly, sequencing studies have also identified rare variants in these three genes that increase the risk for LOAD [35].

#### Replicated genes harboring rare variants with reduced penetrance and contributing to AD risk

In the first study applying ES to AD, we identified a previously known mutation (p.Arg1231Cys) in the notch receptor 3 gene (*NOTCH3*) in a Turkish patient from a consanguineous family, clinically diagnosed with AD. Mutations in this gene, and this specific variant, had been previously shown to cause cerebral autosomal dominant arteriopathy with subcortical infarcts and leukoencephalopathy (CADASIL). Segregation analysis led to identifying the same variant in one unaffected at-risk individual, raising the possibility of incomplete penetrance of the variant in the family. The consanguinity and the spectrum of different phenotypes observed in the family, which included other neurodegenerative and immune diseases, complicated the definite association of this specific variant with AD. Additionally, the resequencing of *NOTCH3* in 95 EOAD patients and 95 controls did not reveal any additional rare *NOTCH3* variants that could be associated with disease [36], and this finding remained inconclusive. In a subsequent study investigating the role of genes known to be causative of adult-onset leukodystrophies in AD, we again identified variants in *NOTCH3* to be associated with AD. We sequenced ten genes in 332 non-Hispanic white AD patients and 676 controls of old age. The gene-based analysis was significant for *NOTCH3,* with the signal being driven by one common synonymous variant (p.Pro1521Pro) and three rare coding variants with large effect sizes (p.Val1952Met, p.Val1183Met, p.His170Arg) [37]. The carrier frequency of the three rare coding variants was 2-3 times higher in LOAD patients when compared to control individuals, and all three variants had previously been significantly associated with severity of white matter lesions in elderly individuals with hypertension [37, 38]. More recently, in a larger study of AD performed by the Alzheimer Disease Sequencing Project (ADSP), more than 5000 AD patients and 4500 controls were tested using ES to identify rare variants associated with the disease. The NOTCH3 p.Ala284Thr was found in 10 AD patients and no controls, further confirming the link between this gene and AD [39].

In 2013, after identifying triggering receptor expressed on myeloid cells 2 (*TREM2*) homozygous mutations as the cause of atypical frontotemporal dementia in three Turkish families [40] we expanded the genetic analyses of this gene to include other dementias. When assessing a cohort of non-Hispanic white AD patients (*n*= 1092) and controls (*n*= 1107), we identified a significantly increased frequency of rare heterozygous variants in cases. The p.Arg47His variant, specifically, showed a strong and significant association with the risk of disease, confirmed by direct genotyping in an extended cohort of 1887 AD patients and 4061 control individuals and a meta-analysis of 3 independent GWAS [41]. A similar finding was made after sequencing the genomes of 2261 Icelanders, conclusively implicating *TREM2* in the pathogenesis of AD [42]. After these studies, associations have been reported across different populations [28], and in a large LOAD family (*n*=21 affected individuals), p.Arg47His was found to co-segregate entirely with the disease [43]. In 2017, it was shown that a second *TREM2* rare coding variant (p.Arg62His) increased the risk for sporadic AD independently of p.Arg47His [11].

Rare variants with a role in AD have also been identified in genes located within loci where common variants had previously been associated with AD risk by GWAS. These include variants in the TP-binding cassette, sub-family A, member 7 (*ABCA7*), in the sortilin-related receptor 1 (*SORL1*), in the Bridging integrator 1 (*BIN1*), and in the Clusterin (*CLU*) genes. In *ABCA7* and *SORL1,* an enrichment in rare variants has been reported in AD patients compared to controls, and rare variants have been identified in families with different degrees of co-segregation with the disease. In both genes, the rare variants associated with disease, which include missense and premature termination codon (PTC) variants, are also found in controls, suggesting variable penetrance.

In *ABCA7,* this enrichment is mainly associated with protein-truncating variants across the transcript and suggests haploinsufficiency as the most likely disease mechanism. Families presenting with an autosomal dominant mode of AD inheritance have been found to carry rare *ABCA7* variants segregating with the disease [44]. De Roeck and colleagues observed incomplete nonsense-mediated decay (NMD) of ABCA7 transcripts harboring PTC variants [45]. For some variants, the induced PTC could be removed from the transcript by using cryptic splice sites. However, alternative splicing only explained variable expression levels in a minimal number of patients.

ES in 14 unrelated EOAD probands of French families identified five carriers of PTCs in *SORL1* [46*]*. This study was, however, unable to test family members due to the unavailability of DNA samples. Still, subsequent studies have confirmed a role for rare variants in this gene in AD [47, 48], particularly for PTCs, again suggesting a haploinsufficiency mechanism [49]. Missense and splice site rare variants in this gene have also been suggested to play a role in autosomal dominant AD, LOAD, and EOAD families with parkinsonian features [50,51,52]. Additionally, bi-allelic loss-of-function of SORL1 was observed in one AD patient with parental history of dementia [53].

In *BIN1*, the rare variant p.Lys358Arg was found to segregate in 2 of 6 Caribbean Hispanic families where the variant was identified. Still, no significant association was found with LOAD in non-Hispanic whites in the same study [54]. The p.Pro318Leu variant was also found to be significantly associated with LOAD in the Han Chinese population [55].

*CLU* was one of the first loci to be identified as associated with AD in GWAS. In addition to common variants in the gene, more recently, rare variants have also been shown to have a role in this disease [56]. This type of variability in *CLU* has, so far, not been described as segregating in AD families. The potential role of rare heterozygous variants in the gene in AD was based on the unbiased resequencing of all *CLU* coding exons and regulatory regions in AD patients and control individuals from Flanders-Belgium. This study identified rare variants (p.Thr345Met, p.Asn369His, p.Thr440Met, p.Arg338Trp, and p.Thr445_Asp447del) clustering in the CLU β-chain domain [56].

Genome-wide meta-analysis using rare variant imputation identified a novel association of a rare variant (rs143080277) in NCK adaptor protein 2 (*NCK2*) with AD [57]. The same variant was identified in a genome-wide meta-analysis, fine-mapping, and integrative prioritization study. It is however difficult to assess how independent the cohorts used in these two studies are, and consequently if this finding is truly independently replicated [58].

The application of ES to study seven African American AD patients with a positive family history identified two rare variants in A-kinase anchor protein 9 (*AKAP9*). These variants (rs149979685 and rs144662445) were also associated with AD risk when comparing cases and controls [59]. Another rare variant in *AKAP9* (p.Arg434Trp) was identified in two large families segregating with the disease [60].

The p.Thr835Met rare coding variant in the Unc-5 homolog C (*UNC5C*), was found to segregate with disease in two families presenting with LOAD and was associated with disease risk in four independent cohorts [61]. Additional rare variants in the gene have been identified in European families (p.Ala860Thr, identified in two families, and p.Pro666Ser) and Chinese cases (p.Gln860His, p.Thr837Lys, p.Ser843Gly, and p.Val836Val) [62, 63].

In 2017, a GWAS using an exome microarray identified genome-wide significant associations between rare variants in *TREM2*, phospholipase C gamma 2 (*PLCG2*), and ABI Family Member 3 (*ABI3*) and AD risk [11]. The PLCG2 variant p.Pro522Arg was a protective variant (discussed more below), while the *ABI3* p.Ser209Phe variant increased the risk for the development of AD [11]. Significant associations of both the *PLCG2* and *ABI3* variants with AD were also observed in other populations [64, 65].

Details as MAF and ancestry for the variants identified in these studies are shown in **Supplementary Table** **1**.

#### Genes harboring rare variants with an initial association to AD

Several new candidate genes have been recently reported to harbor rare variants associated with AD risk or are potential causes of the disease but currently lack consistent support from other studies. Typically small studies have identified these using familial settings or, more recently, by using ES or GS in families and case-control cohorts. For example, by exome sequencing a Jewish Israeli consanguineous family originating from Morocco and clinically diagnosed with early-onset AD, we identified a homozygous *CTSF* mutation (p.Gly415Arg) as the most probable cause of disease in the family [66]. The identification of this variant was based on a previous analysis of extended regions of homozygosity shared by the siblings, but no definite segregation could be established due to the number of samples available for the study [67]. Since these findings, no additional report implicated genetic changes in *CTSF* in AD, which remains an inconclusive association. More recently, and as an example of the use of next-generation sequencing in larger cohorts, Prokopenko and colleagues assessed rare variants by performing single-variant and spatial clustering–based testing in a family-based GS-based association study. This included 2247 subjects from 605 multiplex AD families, followed by replication in 1669 unrelated individuals. They identified 13 novel AD candidate loci that yielded consistent rare-variant signals in discovery and replication cohorts: *FNBP1L, SEL1L, LINC00298, PRKCH, C15ORF41, C2CD3, KIF2A, APC, LHX9, NALCN, CTNNA2, SYTL3*, and *CLSTN2* *[*68*]*. These novel candidate genes will need to be replicated by independent studies to fully establish the association with AD.

An exome-wide age-of-onset analysis revealed a synonymous rare variant (rs56201815) in endoplasmic reticulum to nucleus signaling 1(*ERN1*) to be associated with a higher risk of AD, particularly in *APOE ε4* non-carriers [69].

Other candidate genes are represented in **Table** **1**. From these, it is interesting to note that an exome sequencing study of non-Hispanic white families identified rs137854495 in ATP binding cassette subfamily A member 1 (*ABCA1*) as segregating with disease in one family [70]. This variant was identified under a family-specific linkage peak on chromosome 9, adding evidence to its role in disease.

**Table 1:** Genes/loci harboring rare variants in AD that need independent replication

Gene symbol	Gene	Location	Variant	Ref	Pathway
*AARD*	Alanine and arginine rich domain containing protein	8q24.11	Gene burden	[69]	N.A.
*ABCA1*	ATP binding cassette subfamily A member 1	9q31.1	rs137854495, NM_005502.4:c.2810C>T:p.Ala937Val	[70]	Aβ pathway Lipoprotein metabolism
*ADAM10*	ADAM metallopeptidase domain 10	15q21.3	rs61751103, NM_001110.4:c.510G>C:p.Gln170His rs145518263, NM_001110.4:c.541A>G: p.Arg181Gly	[71]	Aβ pathway Apoptosis, phagocytosis, autophagy
*ADAM17*	ADAM metallopeptidase domain 17	2p25.1	rs142946965, NM_003183.6:c.644G>T:p.Arg215Ile	[72]	Aβ pathway Immune system Apoptosis, phagocytosis, autophagy
*ANXA5*	Annexin A5	4q27	Gene burden	[73]	Immune system
*APC*	APC regulator of WNT signaling pathway	5q22.2	Spatial clustering	[67]	Aβ pathway Axonal pathfinding Synaptic plasticity
*ASRGL1*	Asparaginase and isoaspartyl peptidase 1	11q12.3	chr11:62,343,562:G>C*	[55]	Axonal guidance and cytoskeleton function Apoptosis, phagocytosis, autophagy
*BACE2*	Beta-secretase 2	21q22.2-q22.3	12Kb intronic deletion	[74]	Aβ pathway Apoptosis, phagocytosis, autophagy
*C1orf173/ERICH3*	Glutamate rich 3	1p31.1	Gene burden	[73]	N.A.
*C15ORF41/CDIN1*	CDAN1 interacting nuclease 1	15q14	rs141228575, NM_001130010.3:c.213-2446C>T rs147002962, NM_001130010.3:c.102-13586C>G	[67]	Erythrocyte differentiation
*C2CD3*	C2 domain containing 3 centriole elongation regulator	11q13.4	Spatial clustering	[67]	Cilium assembly Ciliary basal body plasma membrane docking
*CASP7*	Caspase 7	10q25.3	rs116437863, NM_001267057.1:c.1045A>G:p.Arg297Gly	[73]	Aβ pathway Immune system Apoptosis, phagocytosis, autophagy
*CD2AP*	CD2 associated protein	6p12.3	rs116754410, NM_012120.3:c.1898A>G:p.Lys633Arg rs138727736, NM_012120.3: c.1120A>G:p.Thr374Ala	[49]	Immune system Cytoskeleton function BBB integrity Apoptosis, phagocytosis, autophagy
*CD55*	CD55 molecule (Cromer blood group)	1q32.2	chr1:207461994C>T (upstream of *CD55*)	[75]	Immune system
*CLDN17*	Claudin 17	21q21.3	Homozygosity and gene burden	[76]	BBB integrity
*CLSTN2*	Calsyntenin 2	3q23	Spatial clustering	[67]	Morphology of synaptic complexes Calcium-mediated postsynaptic signaling Episodic memory Signal transduction
*CR1*	Complement C3b/C4b receptor 1	1q32.2	rs11587944, NM_000651.6:c.313C>T:p.Arg105Cys rs372477607, NM_000651.6:c.4021C>T:p.Pro1341Ser	[55]	Aβ pathway Immune system Apoptosis, phagocytosis, autophagy
*CSF1R*	Colony stimulating factor 1 receptor	5q32	rs281860278, NM_001288705.3:c.2603T>G:p.Leu868Arg rs748641028, NM_001288705.3: c.2073G>C: p.Gln691His rs111943087, NM_001288705.3:c.2107C>T:p.His703Tyr	[34]	Immune system BBB integrity Cytoskeleton function
*CTNNA2*	Catenin alpha 2	2p12	Spatial clustering	[67]	Neuronal migration and neuritic outgrowth Cell-cell adhesion
*CTSF*	Cathepsin F	11q13.2	rs200426008, NM_003793.4:c.1243G>A: p.Gly415Arg	[65]	Immune system Apoptosis, phagocytosis, autophagy
*DPP6*	Dipeptidyl peptidase like 6	7q36.2	Chr7:149,704,610 - 153,786,893 paracentric inversion*	[77]	Synaptic plasticity
*EPHA1*	EPH receptor A1	7q34-q35	rs202178565, NM_005232.5:c.1379C>T:p.Pro460Leu	[49]	Immune system Axonal guidance Synaptic plasticity BBB integrity Apoptosis, phagocytosis, autophagy
*ERN1*	Endoplasmic reticulum to nucleus signaling 1	17q23.3	rs56201815, NM_001433.5:c.1017C>T:p.Asp339=	[68]	Autophagy Unfolded protein response (UPR) and neuronal apoptosis
*EXOC3L4/C14orf73*	Exocyst complex component 3 like 4	14q32.32	rs117708804, NM_001077594.1:c.1126G>T:p.Ala376Ser rs10142287, NM_001077594.1:c.687C>A:p.Pro229= rs9324055, NM_001077594.1: c.889G>A: p.Val297Met rs148718670, NM_001077594.1: c.1937G>A: p.Arg646Gln	[78]	N.A.
*FERMT2*	FERM domain containing kindlin 2	14q22.1	rs17125944, NM_001134999.1:c.158-14555A>G	[70]	Cell adhesion
*FNBP1L*	Formin binding protein 1 like	1p22.1	rs192471919, NM_001164473.2:c.1652-634T>C	[67]	Endocytosis Autophagy Neurite elongation and axonal branching (neuroplasticity)
*FSIP2*	Fibrous sheath interacting protein 2	2q32.1	rs531170562, NM_173651.4:c.742C>T:p.Arg248Cys rs552474258, NM_173651.4:c.743G>T:p.Arg248Leu	[70]	Spermatogenesis
*IGHG3*	Immunoglobulin heavy constant gamma 3 (G3m marker)	14q32.33	rs77307099, ENST00000390551.2:c.938G>A, p.Ser313Asn rs78376194, ENST00000390551.2: c.939C>T, p.Ser313= rs12890621, ENST00000390551.2: c.674A>T, p.Tyr225Phe	[69]	Neuroinflammation
*KIF2A*	Kinesin family member 2A	5q12.1	Spatial clustering	[67]	Mitotic spindle activity and normal brain development Microtubule-based processes Ciliogenesis Cortical development Spinal cord injuries
*LHX9*	LIM homeobox 9	1q31.3	Spatial clustering	[67]	Development of the forebrain
*LincRNA LINC00298*	Long intergenic noncoding RNA LINC00298	2p25.1	rs147918541, chr2:8073233:G>A	[67]	May be involved in neuronal plasticity
*LncRNA AC099552.4*	Long non-coding RNA AC099552.4	7q36.3	chr7:154988675:G>A	[69]	Gene expression regulation
*LncRNA RP11-433J8*	Long non-coding RNA RP11-433J8	14q32.2	chr14:97228875:A>G	[70]	N.A.
*MAPT*	Microtubule associated protein tau	17q21.31	NM_001123066.3:c.370C>A: p.Gln124Ly NM_001123066.3:c.2203T>G:p.Ser735Ala	[79]	Tau pathway Axonal guidance and cytoskeleton functioning Apoptosis, phagocytosis, autophagy
*MARK4*	Microtubule affinity regulating kinase 4	19q13.32	NM_001199867.1: c.946_951delGGTGAGinsGAT:p.Gly316_Glu317delinsAsp	[74]	Tau pathway Cytoskeleton function Apoptosis, phagocytosis, autophagy
*MYRF*	Myelin regulatory factor	11q12.2	rs34038946, NM_001127392.3:c.2167G>A:p.Ala723Thr	[55]	Lipid metabolism Synaptic plasticity
*NALCN*	Sodium leak channel, non-selective	13q32.3-q33.1	Spatial clustering	[67]	Neuronal excitability
*ND2*	NADH dehydrogenase subunit 2	chrM:4,470-5,511	m.5460G>A and m.5460G>T	[80]	Mitochondrial cascade hypothesis
*PIN1*	Peptidylprolyl cis/trans isomerase, NIMA-interacting 1	19p13.2	c.477C>T, p.Thr152Met*	[81]	Aβ pathway Tau pathway Apoptosis, phagocytosis, autophagy
*PINX1*	PIN2 (TERF1) interacting telomerase inhibitor 1	8p23.1	Gene burden	[82]	Telomere integrity
*PRKCH*	Protein kinase C eta	14q23.1	Spatial clustering	[67]	Signal transduction
*PSD2*	Pleckstrin and Sec7 domain containing 2	5q31.2	rs138380367, NM_032289.4:c.1549G>A: p.Gly517Ser	[83]	Endolysosomal transport
*RTN3*	Reticulon 3	11q13.1	rs372883387, NM_001265589.2:c.-8G>T NM_001265589.2:c.42C>T: p.Ser14= NM_001265589.2: c.116C>T:p.Thr39Met rs11551944: NM_001265589.2:c.17C>A:p.Ala6Glu	[84]	Aβ pathway Lipid metabolism Apoptosis, phagocytosis, autophagy
*RUFY1*	RUN and FYVE domain containing 1	5q35.3	rs138313632,NM_025158.5:c.2113T>G: p.Ser705Ala	[83]	Endolysosomal transport
*SEL1L*	SEL1L adaptor subunit of ERAD E3 ubiquitin ligase	14q31.1	rs74065194, chr14:82182068: C>T	[67]	Aβ pathway Endoplasmic reticulum-associated degradation (ERAD) ER stress and cell death
*SEZ6*	Seizure related 6 homolog	17q11.2	rs371753097, NM_178860.5:c.1844G>A:p.Arg615His	[85]	Aβ pathway Synaptic plasticity
*SLC24A4-RIN3*	Solute carrier family 24 member 4-Ras and Rab interactor 3	14q32.12	SLC24A4:rs10498633, NM_153646.4:c.1255+4000G>T RIN3:rs147042536, NM_024832.5:c.2377T>C, :p.Tyr793His RIN3: rs150221413, NM_024832.5:c.189G>T, :p.Trp63Cys	[55, 83]	SLC24A4: Na+Ca2+, K+ exchange RIN3:Endolysosomal transport
*SRCAP*	Snf2 related CREBBP activator protein	16p11.2	10 ultra-rare missense mutations	[86]	Gene expression regulation
*SYTL3*	Synaptotagmin like 3	6q25.3	Spatial clustering	[67]	Vesicle trafficking
*TCIRG1*	T cell immune regulator 1, ATPase H+ transporting V0 subunit a3	11q13.2	rs34227834, NM_006019.4:c.482C>T, : p.Pro161Leu	[83]	Endolysosomal transport Immune system
*tRNAGLN*	Transfer ribonucleic acid glutamine	chrM	m.4336A>G	[61]	Mitochondrial cascade hypothesis
*TTC3*	Tetratricopeptide repeat domain 3	21q22.13	rs377155188, NM_001001894.2:c.3113C>G: p.Ser1038Cys	[87]	Aβ pathway
*TYROBP*	Transmembrane immune signaling adaptor TYROBP	19q13.12	rs200649978, NM_003332.4:c.5G>A:p.Gly2Glu NM_003332.3:c.163G>T: p.Val55Leu	[88]	Aβ pathway Immune system Lipid metabolism Apoptosis, phagocytosis, autophagy
*VPS35*	VPS35 retromer complex component	16q11.2	NM_018206.6:c.1874T>C: p.Leu625Pro	[74]	Aβ pathway Apoptosis, phagocytosis, autophagy
*VWA2*	Von Willebrand factor A domain containing 2	10q25.3	rs79009215, NM_001272046.2:c.1096G>A:p.Val366Met	[89]	N.A.
*ZNF655*	Zinc finger protein 655	7q22.1	Gene burden	[69]	Gene expression regulation

Genes and loci reported to harbor rare variants that have been associated with AD, their chromosomal locations and respective biological pathways and processes. Variants shown here represent the original associations established with AD in the respective studies. When the findings were focused on gene or regional burden analyses, that is noted, instead of specific variants. *As reported in the original publication. AD, Alzheimer’s disease; Aβ, Amyloid-beta; BBB, Blood-Brain Barrier; ERAD, Endoplasmic reticulum-associated degradation; ER stress, Endoplasmic reticulum stress; UPR, Unfolded protein response.

**Fig. 1:**
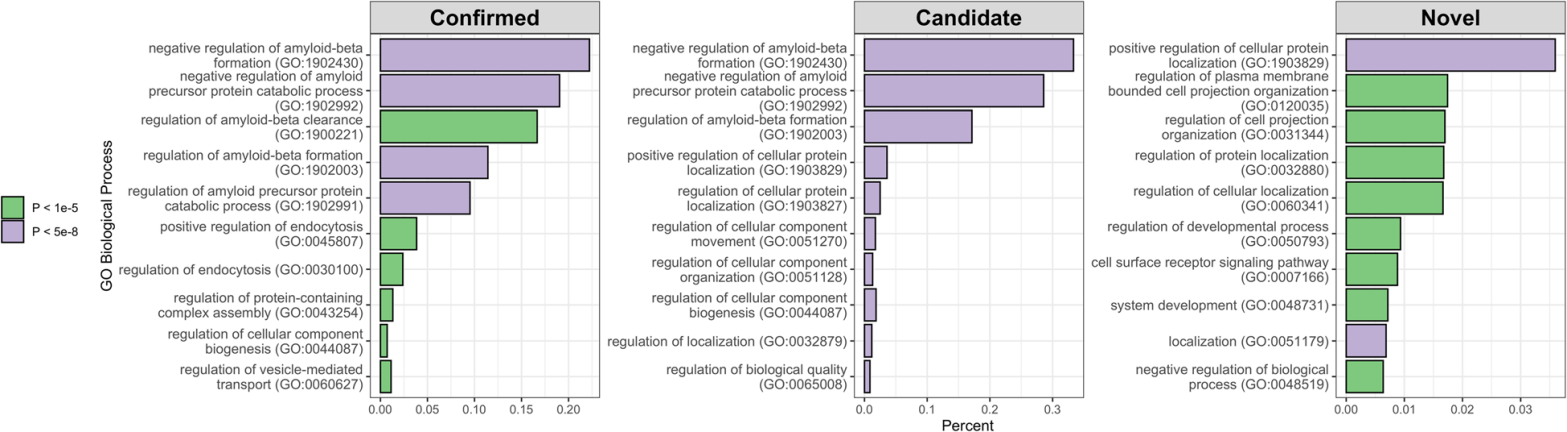
Top results (based on Fisher's Exact test p-value) for Confirmed (genes with strong evidence or replication) or Candidate (suggestive role, replication needed) in AD, based on the genes described in this review. Also shown are pathways emerging only with the addition of these Candidate genes (Novel). Gene set analyses were performed using the Gene Ontology website (http://geneontology.org/), analyzing the provided biological processes of the gene set. The X-axis represents the percent of the pathway represented out of total genes possible for that pathway. Y-axis shows gene ontology terms and IDs. The color of the bars indicates the significance level.

Processing of amyloid-beta emerges as the main biological pathway associated with AD when analyzing gene sets harboring rare variants. This is true for genes confirmed to harbor rare variants associated with AD and for genes that have rare variants with an initial association with AD, that need to be independently replicated (Fig. 1). This is interesting to note as APP metabolism has mainly been associated with AD using pathway analyses when common variants were assessed by GWAS in very large sample sizes, and confirms the nominal association for the amyloid-beta pathway obtained by Kunkle et al. when using rare variants [21, 71].

It will be very challenging to replicate these candidate variants and genes in truly independent cohorts, given the sample size needed to detect and associate most of these rare variants. Resources like the UK Biobank will be valuable in these efforts and functional follow up of the findings will be important to understand the true roles of these variants in disease.

#### Rare protective variants

Rare protective variants are of high interest as they can be used as a model for the development of drugs mimicking their effects.

In 2014 Medway and colleagues investigated rare variants within the *APOE* locus and identified the rare haplotype *APOEε3b* harboring the p.Val236Glu variant on the ε3 background and significantly associated with a decrease in AD risk comparable to that of the *APOE ε2* allele [72]. More recently, an individual from a large Colombian AD kindred carrying the pathogenic *PSEN1* p.Glu280Ala mutation was described to be remarkably resistant to the clinical onset of autosomal dominant AD dementia. ES confirmed the presence of the *PSEN1* p.Glu280Ala mutation and revealed two copies of the rare *APOEε3* p.Arg136Ser variant. The potential protective effect of the *APOEε3* p.Arg136Ser variant on disease onset, suggests this variant can be a model for the development of novel therapies [73].

In the same Icelandic population where *TREM2* p.Arg47His was identified as a risk factor for AD, Jonsson and colleagues also identified a rare variant in *APP* (rs63750847, p.Ala637Thr) with a MAF of 0.01%, that was significantly more common in elderly healthy individuals than in AD subjects, indicative of a protective effect against the development of AD. Additionally, the protective allele was found to be associated with increased performance on cognitive tests in elderly cognitively normal participants, suggesting the protective effect of the variant is not limited to AD pathogenesis but also affects cognition in individuals within the healthy spectrum of aging [74].

The previously mentioned protective variant (rs72824905, p.Pro522Arg) identified in *PLCG2*, the gene encoding the enzyme phospholipase-C-γ2 that is highly expressed in microglia, was found to be associated with 1.5-fold reduced risk of AD in a three-stage case-control study of 83,133 subjects of European ancestry. The variant is located in a regulatory domain, conferring a small hypermorphic effect on enzyme activity [75] and has been shown to enhance microglial functions in a Plcγ2-P522R knock-in mouse model [76]. PLCγ2 regulates microglial functions via TREM2-dependent and -independent signaling pathways being potentially involved in the transition to a microglial state associated with neurodegenerative disease [77]. This variant was also shown to reduce AD disease progression by mitigating tau pathology in the presence of amyloid pathology. The protective effect was more pronounced in MCI patients with low Aβ1-42 levels, suggesting a role of PLCG2 in the response to amyloid pathology [78]. Adding to the notion that p.Pro522Arg exerts its strongest effect downstream of amyloid pathology, Sierksma and colleagues also found PLCG2 as part of a unique gene expression module specifically responsive to Aβ but not TAU pathology [79]. Altogether, these findings support the idea that a weak lifelong activation of the PLCG2 pathway may confer protection against AD, and pharmacological modulation of microglia via TREM2-PLCγ2 pathway-dependent stimulation may be a novel therapeutic option for the treatment of AD.

Even though no individual rare variants in the *MS4A* gene cluster and *ABCA1* have reached consistent statistical significance in association with the risk of AD, a higher, nominally significant burden of rare coding variants has been found in controls compared to AD in both cases. Screening of the *MS4A* gene cluster in 210 AD cases and 233 controls identified missense and loss-of-function variants twice as frequently in controls than cases [80]. Similarly, a rare variant candidate gene study sequencing 311 LOAD cases and 360 controls found that 1.7 times as many controls had at least one rare variant (MAF < 0.05) in *ABCA1* than cases [81]. This suggests that similarly to *APP*, rare variability in this gene may be associated with both protective and causative roles in AD.

## The challenges when studying rare variants

Identifying rare variants and their subsequent association with disease risk is much more complex and less powerful than for common variants. Several factors contribute to this, mainly related to the methodologies used to detect and analyze variants.

An example of how difficult it is to assess the true association of rare variants with a specific disease comes from the description of Phospholipase D3 gene (*PLD3*) as a risk gene for AD. Using ES, Cruchaga and colleagues studied 14 LOAD families and identified a rare variant (p.Val232Met) in *PLD3* segregating with the disease in two of these families. In the same report, the subsequent analyses in large case-control cohorts also showed a significant association between the variant and AD, as did the burden analysis for the gene. PLD3 was also shown to be involved in amyloid-β precursor protein processing and was found to be overexpressed in brain tissue from patients with AD [82]. More recently, a study using human brain tissue and mouse models has suggested that PLD3 has an important role in AD through lysosomal dysfunction. Nackenoff A. et al. established PLD3 as a lysosomal phospholipase D and showed that the AD-associated variant identified by Cruchaga and colleagues impaired its function. PLD3 expression levels were also found to correlate with β-amyloid plaque density and the rate of cognitive decline in humans. Similarly, PLD3 expression levels correlated with memory and learning in a genetically diverse mouse model [83]. However, since the initial association of *PLD3* with AD, independent studies with sufficient power to detect similar genetic effects have all failed to replicate the association [84–88]. This lack of statistical evidence for association in independent cohorts highlights the complexity of establishing rare variants as contributors to complex diseases. In this case, it may be due to different scenarios: it may indicate that the initial association was actually a false positive, it may represent such a small effect that the signal is not consistently identifiable in other cohorts, or it can reflect a genetic influence only in familial settings. In the latter scenario, it would represent an effect in very rare families, as additional mutations in the gene have not been identified so far in other AD families.

### Methodological challenges

As previously mentioned, rare variants are typically detected and assessed using either microarrays or next-generation sequencing technologies. GS is the only methodology that virtually assesses the totality of variability in one sample. Microarrays will only test known variants included in the array’s design, and ES will generally not detect non-coding variants. When working with genotyping calls of rare variants, it is often needed to confirm the genotyping clusters visually, and when working with GS data, it is usually necessary to have access to high power computing. This is particularly true when performing analyses of GS data in the large numbers of samples required for meaningful associations of rare variants with disease (see next section). Given the cost and computing needs, most sequencing studies aimed at detecting rare variants have used ES. To increase the study’s sample size, it has been common to merge data from different sources and/or perform targeted sequencing. While this is relatively straightforward when performing GWAS using data from genotyping arrays, it is more complex when merging data generated using different exome capture methods and with varying coverage [89]. Sequence capture uniformity and capture probe performance will determine how much raw sequence data for each sample will be available and, consequently, impact downstream analyses [90].

#### Sample sizes

When studying rare variants in the context of disease, sample sizes are critical for detecting variants and establishing their association with disease risk. Even before establishing an association with disease, it is already challenging to detect rare variants: it is necessary to study at least 460 or 4600 individuals to detect alleles, with a probability of 99%, with frequencies of 0.5% or 0.05%, respectively [91]. To obtain sufficient statistical power, the association of rare variants with risk of disease requires larger sample sizes than those needed to associate common variants. For example, when the effect size of a variant is 0.1 phenotyping standard deviation units (corresponding to an odds ratio of ~1.2), a common variant with MAF = 10% needs ~10,000 individuals to obtain genome-wide significance at *P* = 5 × 10−8 with 80% statistical power. Variants with MAFs of 1% and 0.1% require ~100,000 and 1 million individuals, respectively [92]. It is commonly accepted that a well-powered rare variant association study should involve discovery sets with at least 25,000 cases, and a substantial replication set [93]. Consequently, the typical GWAS approach of analyzing one variant at a time is typically underpowered for rare variants unless the variant effect is substantial. Methods collapsing several rare variants together have been developed to overcome this, but these still require large sample sizes. This is the case of the latest large gene burden study encompassing a total of 32,558 samples (16,036 AD cases and 16,522 controls) that led to the identification of 11 genes associated with AD-risk, of which rare variants in eight genes were not previously significantly associated with the disease [94].

One way to overcome the challenge of large sample sizes is to study isolated or distinct populations. An ultra-rare variant in one population may be more frequent in another population. A prime example is the *APP* p.Ala637Thr variant previously mentioned. This variant is virtually absent in Asian and African populations, has a MAF of 0.0003 in European non-Finish populations and of 0.003 in the Finnish. This distribution of frequencies makes it very unlikely that the finding of its protective role in AD could have been done outside Iceland.

#### Impact and interpretation of rare variants

The analyses of ever larger sample sizes and the development of improved statistical methods lead to increased statistical power in the association of rare variants with AD risk. At the same time, the study of well-characterized and carefully selected families can also result in the identification of rare variants with significant roles in the pathogenesis of AD. However, both in case-control association studies and in familial settings, an important limiting factor continues to be assessing the impact of the variants and their interpretation in the context of disease.

The development of *in silico* prediction software and the active cataloging of rare variants in publicly available databases have contributed significantly to interpreting the effects of rare variants in genes and proteins. These are particularly useful, given the sheer number of variants typically detected in an individual exome or genome. For example, CADD is a popular tool used to predict the pathogenicity of clinical variants. It uses a logistic regression model and dozens of genomic features to learn the characteristics of randomly generated variants and distinguish these from recently fixed variants in humans [95]. However, this type of predictors is limited by the data available for most genes, including the number of known pathogenic variants and associated functional data. More recently, machine learning techniques such as deep learning have been used for genome functional annotation and assessment of variant function [96]. With better computer power and the increase in the amount of data available, this type of approach will allow for more and better results. And in cases with limited data, as often occurs for poorly studied genes and ultra-rare variants, alternative approaches such as transfer learning can be applied to study rare variants by using the information on genes similar to the gene of interest, for example [97].

It is crucial to keep in mind that a variant that damages a gene or protein is not necessarily damaging in terms of health and disease [98]. The interpretation of pathogenicity of rare variants and the understanding of their relevance to disease needs the integration of diverse data. Essential factors to consider include the prevalence and mode of inheritance of the disease. Also, genetic heterogeneity, reduced penetrance and variable expressivity of the variant, composite phenotypes, pleiotropy, and epistasis should all be taken into account [99].

Variant interpretation refers to the process of connecting individual variants to disease phenotypes. This is a complex process that may have a significant impact on a patient's diagnosis or treatment. The guidelines developed by the American College of Medical Genetics (ACMG) provided a systematic way of understanding the clinical significance of any given sequence variant and have set the standards for this process [100]. Still, this continues to be a challenging process dependent on expert interpretation based on literature review. These guidelines are general, and there is an imminent need to establish procedures and databases specific to different genes and diseases.

### The ultimate challenge: rare non-coding variants

As previously mentioned, the vast majority of variants identified as associated with AD risk by GWAS are located in non-coding regions of the genome. It has been challenging to pinpoint the true functional variant(s) at these loci and, most importantly, understand how these changes influence the molecular mechanisms and risk of disease [101]. To accomplish this, fine-mapping and expression quantitative trait loci (eQTL)-based approaches have been used to identify multiple candidate causal genes, and have demonstrated significant associations between AD risk and gene expression. It is, thus, critical to have detailed information from functional mapping with the identification of regulatory elements, causal cell types/tissue(s), genes, and pathways. These approaches provide insight into likely mechanisms of actions of candidate genes for further functional validation in cell and animal models [102].

When analyzing GS data, it is common to discard this type of variability to streamline the data analysis process. This is done because the probability of being able to discern a true effect of a rare non-coding variant in disease is low, and because of the need to reduce the number of variants to be analyzed in detail. Nonetheless, the Encyclopedia of DNA Elements (ENCODE) project emphasized that as much as 80% of the non-protein-coding portion of the genome is associated with biochemical ‘function’ [103], clearly highlighting the importance of assessing non-coding variability in AD and other diseases. Efforts like the Atlas of Variant Effect Alliance are working toward this end with the major goal of interpreting the impact of all genomic variation. These initiatives are essential as non-coding variant prioritization tools are less accurate than their protein-coding counterparts and there is currently insufficient understanding of the regulatory machinery encrypted in non-coding DNA [98].

Some recent studies have attempted to incorporate non-coding information into burden analyses when studying complex diseases, including AD. Examples are the identification of *CAV1* as an ALS risk gene, after performing a burden analysis for rare variants within enhancers [104], and the identification of 5 loci containing rare alleles with a substantial contribution to the heritability of type 2 diabetes, using islet annotation to create a non-coding framework for rare variant aggregation testing [105].

In AD (and other neurodegenerative diseases) non-coding and loss-of-function coding variants in *TET2* were associated with disease [106]. These results need to be independently replicated, particularly given the potential somatic origin of *TET2* mutations, and the disparity in age between cases and controls used [107].

## Conclusion

Advances in genetic technology and analysis methods have led to a faster pace in the identification of rare genetic variability as the cause of AD or associated with an increase or decrease of its risk. As a result, rare variants such as those in *TREM2, SORL1,* and *ABCA7* are now well established. However, many rare variants identified in novel genes are still waiting to be replicated. This is challenging given the sample sizes required and the need for independent replication cohorts.

The ultimate goal of understanding genetic risk mechanisms is to translate genetic association to novel drug targets and therapeutics to prevent or delay the onset of disease. Drug discovery and development for a complex disease like AD are exceptionally challenging. This challenge is compounded by an incomplete understanding of AD pathogenesis, the multifactorial etiology and complex pathophysiology of the disease, and importantly, by the lack of good *in vivo* models of AD to translate knowledge from genetics to drugs. Nelson and colleagues showed that genes associated with variation in human traits have provided more targets for successful therapeutic drugs than those without such links. They also suggested that well-studied genes known to be associated with disease proceed better in the drug development pipeline. In addition, they estimate that the success rate in clinical development could be doubled by selecting genetically supported targets [108]. In conclusion, drug development can be facilitated by genetic and genomic knowledge. A complex disease like AD will most likely not be treated by one individual drug. Having multiple targets that allow the development of drugs acting in different steps of the multiple biological pathways involved in AD will probably be a viable approach. For this to happen in the near future we need to continue detailing the genetic architecture of AD. Potential ways forward will most likely include improved methods of simultaneous assessing common and rare variants; the integration of several layers of information such as expression, methylation, biological pathways, and clinical data; and the inclusion of data from publicly available databases and large datasets such as the UK Biobank, Genomics England and gnomAD.

The continuous sharing of data and resources will be the only way to fully understand the biological mechanisms, enable drug development and advance the clinical diagnosis and disease management of such a complex disease as AD.

## Supplementary Information

**Additional file 1.**

## Data Availability

Not Applicable.

## Abbreviations

AD: Alzheimer’s disease
Aβ: Amyloid-beta
ADSP: Alzheimer Disease Sequencing Project
ACMG: American College of Medical Genetics
BBB: Blood-Brain Barrier
CADASIL: Cerebral autosomal dominant arteriopathy with subcortical infarcts and leukoencephalopathy
EOAD: Early-onset AD
ERAD: Endoplasmic reticulum-associated degradation
ER stress: Endoplasmic reticulum stress
eQTL: Expression quantitative trait loci
ENCODE: Encyclopedia of DNA Elements
GWAS: Genome-wide association studies
LOAD: Late-onset AD
MAF: Minor allele
MCI: Mild cognitive impairement
NGS: Next-generation sequencing
NMD: Nonsense-mediated decay
OMIM: Online Mendelian Inheritance in Man
PTC: Premature termination codon
PRS: Polygenic risk scores
SNVs: Single nucleotide variants
UPR: Unfolded protein response
ES: Exome sequencing
GS: Genome sequencing
