# Correction: Challenge accepted: Uncovering the role of rare genetic variants in Alzheimer’s disease

**DOI:** 10.1186/s13024-022-00575-3

**Published:** 2022-11-01

**Authors:** Marzieh Khani, Elizabeth Gibbons, Jose Bras, Rita Guerreiro

**Affiliations:** 1 School of Biology, College of Science, University of Tehran, Tehran, Iran; 2 Department of Neurodegenerative Science, Van Andel Institute, 333 Bostwick Ave. N.E., Grand Rapids, 49503-2518 Michigan, USA; 3 Division of Psychiatry and Behavioral Medicine, Michigan State University College of Human Medicine, Grand Rapids, MI USA

**Molecular Neurodegeneration (2021) 17:3**

10.1186/s13024-021-00505-9

The original article [1] contained an error in the correct assignment of citations within the article to their respective references which has since been corrected.

