# Supplementary material for: Challenge accepted: uncovering the role of rare genetic variants in Alzheimer’s disease
Source: Mol Neurodegener. 2022 Jan 9;17:3. doi: 10.1186/s13024-021-00505-9 (PMC8744312; doi:10.1186/s13024-021-00505-9)
Supplement: Supplementary file 1 — Additional file 1. [file 13024_2021_505_MOESM1_ESM.docx]

**Supplementary Table 1. Rare variants with reduced penetrance and contributing to AD risk, identified in replicated genes**

| **Gene** | **Variant** | **MAF (TOPMED, 1000Genomes, ExAC)** | **Ancestry/ Origin** | **Ref** |
| --- | --- | --- | --- | --- |
| *NOTCH3* | rs201680145, NM_000435.3:c.3691G>A, p.Arg1231Cys | A=0.000204/54 (TOPMED), A=0.000799/4 (1000Genomes), A=0.001004/120 (ExAC) | Turkish | [[1]](https://paperpile.com/c/GWUdu2/JFHH) |
|  | rs1044006, NM_000435.3: c.4563A>G, p.Pro1521Pro    rs115582213, NM_000435.3:c.5854G>A, p.Val1952Met    rs10408676, NM_000435.3:c.3547G>A, p.Val1183Met    rs147373451, NM_000435.3:c.509A>G, p.His170Arg | A=0.103842/27486 (TOPMED), A=0.128195/642 (1000Genomes), A=0.118757/10491 (ExAC)    A=0.007152/1893 (TOPMED), A=0.004992/25 (1000Genomes), A=0.008494/1029 (ExAC)    A=0.085927/22744 (TOPMED), A=0.084465/423 (1000Genomes), A=0.02996/3585 (ExAC)    G=0.001564/414 (TOPMED), G=0.001398/7 (1000Genomes), G=0.001917/224 (ExAC) | NHW | [[2]](https://paperpile.com/c/GWUdu2/n9sd) |
|  | rs149307620, NM_000435.3:c.850C>T, p.Ala284Thr | T=0.000238/63 (TOPMED), T=0.000302/35 (ExAC) | European | [[3]](https://paperpile.com/c/GWUdu2/cf0c) |
| *TREM2* | rs75932628, NM_018965.4:c.140C>T, p.Arg47His | T=0.002/10 (1000Genomes) | NHW | [[4]](https://paperpile.com/c/GWUdu2/CLlY) |
|  | rs75932628, NM_018965.4:c.140C>T, p.Arg47His | T=0.002/10 (1000Genomes) | Icelanders | [[5]](https://paperpile.com/c/GWUdu2/tpwt) |
|  | rs75932628, NM_018965.4:c.140C>T, p.Arg47His | T=0.002/10 (1000Genomes) | White European &Japanese | [[6]](https://paperpile.com/c/GWUdu2/iQgO) |
|  | rs143332484, NM_018965.4:c.185C>T, p.Arg62His | T=0.004992/25 (1000Genomes), T=0.008265/1002 (ExAC) | NA | [[7]](https://paperpile.com/c/GWUdu2/GJAL) |
| *ABCA7* | rs547447016, c.2124_2130del7, p.Glu709fs*    rs376824416, NM_019112.4:c.3578-2A>G    rs142076058, NM_019112.4:c.1732_1775del, p.Arg578Alafs*    rs538591288, NM_019112.4:c.4208del, p.Leu1403fs* | -=0.001621/429 (TOPMED), -=0.000599/3 (1000Genomes), -=0.002413/97 (ExAC)    G=0.000106/28 (TOPMED)    -=0.018371/92 (1000Genomes), -=0.010756/251 (ExAC)    -=0.000884/234 (TOPMED), -=0.001104/125 (ExAC) | Belgian  NHW  African Americans  German | [[8]](https://paperpile.com/c/GWUdu2/sYZV) |
|  | rs199517248, NM_019112.4:c.67-1G>A    NM_019112.4:c.124_130dup, p.His44fs*    NM_019112.4:c.206G>A, p.Trp69*    NM_019112.4:c.302+1G>C    NM_019112.4:c.579+1G>T    NM_019112.4:c.1109dup, p.Met370fs*    NM_019112.4:c.1968_1977del, p.Cys659fs*    rs547447016, NM_019112.4:c.2126_2132del, p.Glu709fs*    NM_019112.4:c.2134G>T, p.Glu712*    rs1030634619, NM_019112.4:c.2194C>T, p.Gln732*    rs774995833. NM_019112.4:c.2544del, p.Thr849fs*    rs373195428, NM_019112.4:c.3577+1G>C    NM_019112.4:c.4008G>A, p.Trp1336*    rs538591288, NM_019112.4:c.4208del, p.Leu1403fs*    NM_019112.4:c.4382G>A, p.Trp1461*    rs113809142, NM_019112.4:c.4416+2T>G    rs753664323, NM_019112.4:c.4465C>T, p.Arg1489*    rs200538373, NM_019112.4:c.5570+5G>C | A=0.000223/59 (TOPMED), A=0.0002/1 (1000Genomes), A=0.000124/15 (ExAC)    -    -    -    -    -    -    -    -    T=0.000011/3 (TOPMED)    -=0.00011/29 (TOPMED), -=0.000053/6 (ExAC)    C=0.000019/5 (TOPMED), C=0.000008/1 (ExAC)    -    -=0.000884/234 (TOPMED), -=0.001104/125 (ExAC)    -    G=0.000246/65 (TOPMED), G=0.000156/18 (ExAC)    T=0.000045/12 (TOPMED), T=0.000067/8 (ExAC)    C=0.000998/5 (1000Genomes), C=0.002579/287 (ExAC) | European | [[9]](https://paperpile.com/c/GWUdu2/TEls) |
| *SORL1* | NM_003105.6:c.422A>G, p.Tyr141Cys    NM_003105.6:c.1531G>C, p.Gly511Arg    rs377498269, NM_003105.6:c.2771A>G, p.Asn924Ser    rs747306346, NM_003105.6:c.4073A>G, p.Asn1358Ser    NM_003105.6:c.4434C>A, Cys1478*    rs1565352546, NM_003105.6:c.5042G>A, p.Gly1681Asp    rs1400205181, NM_003105.6:c.5463G>A, p.Trp1821* | -    -    G=0.000038/10 (TOPMED), G=0.000041/5 (ExAC)    G=0.000019/5 (TOPMED    G=0.000041/5 (ExAC)    -    - | French | [[10]](https://paperpile.com/c/GWUdu2/YjVV) |
|  | NM_003105.6:c.1211+2T>G    NM_003105:c.162delC, p.Asp54fs*^&^    NM_003105:c.1938delA, p.Gly646fs*^&^    NM_003105.6:c.2603del, p.Thr868fs*    rs1480691710, NM_003105.6:c.802C>T, p.Arg268*    NM_003105.6:c.3647G>A, p.Trp1216*    NM_003105.6:c.4434C>A, p.Cys1478*    rs1400205181, NM_003105.6:c.5463G>A, p.Trp1821*    NM_003105.6:c.6163G>T, p.Glu2055*    NM_003105.6:c.372C>G, p.Ser124Arg    NM_003105.6:c.422A>G, p.Tyr141Cys    rs772110877, NM_003105.6:c.994C>T, p.Arg332Trp    NM_003105.6:c.1418G>C, p.Cys473Ser    NM_003105.6:c.1531G>C, p.Gly511Arg    NM_003105.6:c.1628G>A, p.Gly543Glu    NM_003105.6:c.1960C>T, p.Arg654Trp    rs1555071970, NM_003105.6:c.2185C>T, p.Arg729Trp    rs1434306949, NM_003105.6:c.2416G>A, p.Asp806Asn    NM_003105.6:c.3656A>G, p.Asp1219Gly    rs201196740, NM_003105.6:c.3727C>T, p.Arg1243Cys    NM_003105.6:c.4166A>T, p.Asp1389Val    NM_003105.6:c.4961C>T, p.Pro1654Leu    rs1565352546, NM_003105.6:c.5042G>A, p.Gly1681Asp    rs140327834, NM_003105.6:c.6194A>T, p.Asp2065Val | -    -    -    -    -    -    -    -    -    -    -    T=0.000019/5 (TOPMED), T=0.000058/7 (ExAC)    -    -    -    -    T=0.000008/1 (ExAC)    A=0.000004/1 (TOPMED)    -    T=0.000026/7 (TOPMED), T=0.0002/1 (1000Genomes), T=0.000008/1 (ExAC)    -    -    -    T=0.002206/584 (TOPMED), T=0.000799/4 (1000Genomes), T=0.002823/338 (ExAC) | French | [[11]](https://paperpile.com/c/GWUdu2/CjNg) |
|  | NM_003105.6:c.1050_1057del, p.Tyr350fs*    rs144585461, NM_003105.6:c.1246C>T, p.Arg416*    g.68579_68579insA, c.1338insA, p.Gly447Argfs*^&^    g.93142_93148delCCCCATG, c.1966_1972delCCCCATG, p.Thr659Serfs*^&^    NM_003105.6:c.2253_2254del, p.Cys752Serfs*    NM_003105.6:c.3306del, p.Cys1103Valfs*    rs1469648095, NM_003105.6:c.4324C>T, p.Arg1442*    g.155974_155974delG, c.5241delG, p.Val1747 fs* | -    -    -    -      -    -    -    - | Spain    Sweden    Belgium    Spain      Italy    Spain    Portugal    Belgium | [[12]](https://paperpile.com/c/GWUdu2/bqdd) |
|  | rs117260922, NM_003105.6:c.808G>A, p.Glu270Lys    rs143571823, NM_003105.6:c.2840C>T, p.Thr947Met | A=0.015052/3984 (TOPMED), A=0.007788/39 (1000Genomes), A=0.014682/1782 (ExAC)    T=0.003395/17 (1000Genomes), T=0.001277/155 (ExAC) | Caribbean Hispanics | [[13]](https://paperpile.com/c/GWUdu2/fmCb) |
|  | rs752726649, NM_003105.6:c.1763C>T, p.Thr588Ile    rs142884576, NM_003105.6:c.6401C>T, p.Thr2134Met | T=0.000011/3 (TOPMED), T=0.000008/1 (ExAC)    T=0.000599/3 (1000Genomes), T=0.000222/27 (ExAC) | NHW | [[14]](https://paperpile.com/c/GWUdu2/1mjv) |
|  | rs781023219, NM_003105.6:c.3907C>T, p.Arg1303Cys    NM_003105.6:c.3050-2A>G, p.Gly1017- Glu1074del    rs777194720, NM_003105.6:c.5195G>C, p.Gly1732Ala | T=0.000033/4 (ExAC)    -    C=0.000004/1 (TOPMED), C=0.000041/5 (ExAC) | European    European    Swedish | [[15]](https://paperpile.com/c/GWUdu2/DUTN) |
|  | NM_003105.6:c.1211+2T>G    c.3947-3insG^&^    rs200889461, NM_003105.6:c.4265A>G, p.Asn1422Ser | -    -    G=0.00003/8 (TOPMED),G=0.000025/3 (ExAC) | French | [[16]](https://paperpile.com/c/GWUdu2/4opf) |
| *BIN1* | rs138047593, NM_139343.3:c.1625T>C, p.Lys358Arg | C=0.007103/1880 (TOPMED), C=0.002995/15 (1000Genomes), C=0.011219/1360 (ExAC) | Caribbean Hispanic | [[17]](https://paperpile.com/c/GWUdu2/099O) |
|  | rs754834233, NM_139343.3:c.953C>T, p.Pro318Leu | T=0.000058/2 (ExAC) | Han Chinese | [[18]](https://paperpile.com/c/GWUdu2/YPnH) |
| *CLU* | rs563432916, ENST00000560366.1:c.1034C>T, p.Thr345Met    rs9331936, ENST00000560366.1:c.1105A>C, p.Asn369His    rs375247155, ENST00000560366.1:c.1319C>T, p.Thr440Met    rs143634423, ENST00000560366.1:c.1012C>T, p.Arg338Trp    NM_001831.4:c.1333_1341del, p.Thr445_Asp447del | -    C=0.069957/18517 (TOPMED), C=0.065296/327 (1000Genomes), C=0.019224/2329 (ExAC)    T=0.00003/2 (ExAC)    T=0.000015/4 (TOPMED)    - | Flanders-Belgium | [[19]](https://paperpile.com/c/GWUdu2/pPwZ) |
| *NCK2* | rs143080277, NM_003581.5:c.-201+4461T>C | C=0.003177/841 (TOPMED), C=0.001398/7 (1000Genomes) | NA | [[20]](https://paperpile.com/c/GWUdu2/3Rts) |
|  | rs143080277, NM_003581.5:c.-201+4461T>C | C=0.003177/841 (TOPMED), C=0.001398/7 (1000Genomes) | NA | [[21]](https://paperpile.com/c/GWUdu2/Tv87) |
| *AKAP9* | rs144662445, NM_005751.5:c.7638A>G, p.Ile2546Met    rs149979685, NM_005751.5:c.11300C>T, p.Ser3767Leu | G=0.002607/690 (TOPMED), G=0.001597/8 (1000Genomes), G=0.00063/76 (ExAC)    T=0.001572/416 (TOPMED), T=0.001398/7 (1000Genomes), T=0.000432/52 (ExAC) | African American | [[22]](https://paperpile.com/c/GWUdu2/Xsho) |
|  | rs771608420, NM_005751.5:c.1300C>T, p.Arg434Trp | T=0.000038/10 (TOPMED) | Caribbean Hispanic | [[23]](https://paperpile.com/c/GWUdu2/i4j6) |
| *UNC5C* | rs137875858, NM_003728.4:c.2504C>T, p.Thr835Met | T=0.000321/85 (TOPMED), T=0.00033/40 (ExAC) | NA | [[24]](https://paperpile.com/c/GWUdu2/ci0S) |
|  | rs372767649, NM_003728.4:c.2580G>C, p.Gln860His    rs368284839, NM_003728.4:c.2510C>A, p.Thr837Lys    rs779272234, NM_003728.4:c.2527A>G, p.Ser843Gly    NM_003728.4:c.2508C>G, p.Val836Val | C=0.000049/13 (TOPMED), C=0.000599/3 (1000Genomes), C=0.00014/17 (ExAC)    -    G=0.000023/6 (TOPMED), G=0.000074/9 (ExAC)    - | Chinese | [[25]](https://paperpile.com/c/GWUdu2/vM72) |
|  | rs34585936, XM_005263321.3:c.2578C>T, p.Ala860Thr^&^    rs760453427, ENST00000513796.1:c.1996G>A, p.Pro666Ser^&^ | T=0.019461/5151 (TOPMED), T=0.018371/92 (1000Genomes), T=0.018594/2256 (ExAC)    A=0.000004/1 (TOPMED), A=0.000008/1 (ExAC) | European | [[26]](https://paperpile.com/c/GWUdu2/QLN2) |
| *PLCG2* | rs72824905, NM_002661.5:c.1565C>G, p.Pro522Arg | G=0.002796/14 (1000Genomes), G=0.005076/607 (ExAC) | NA | [[7]](https://paperpile.com/c/GWUdu2/GJAL) |
|  | rs72824905, NM_002661.5:c.1565C>G, p.Pro522Arg | G=0.002796/14 (1000Genomes), G=0.005076/607 (ExAC) | European | [[27]](https://paperpile.com/c/GWUdu2/QzhR) |
|  | rs72824905, NM_002661.5:c.1565C>G, p.Pro522Arg | G=0.002796/14 (1000Genomes), G=0.005076/607 (ExAC) | Caucasian | [[28]](https://paperpile.com/c/GWUdu2/Ngwg) |
| *ABI3* | rs616338, NM_016428.3:c.626A>T, p.Ser209Phe^&^ | T=0.00544/1440 (TOPMED), T=0.001597/8 (1000Genomes), T=0.005712/212 (ExAC) | NA | [[7]](https://paperpile.com/c/GWUdu2/GJAL) |
|  | rs616338, NM_016428.3:c.626A>T, p.Ser209Phe^&^ | T=0.00544/1440 (TOPMED), T=0.001597/8 (1000Genomes), T=0.005712/212 (ExAC) | European | [[27]](https://paperpile.com/c/GWUdu2/QzhR) |
|  | rs616338, NM_016428.3:c.626A>T, p.Ser209Phe^&^ | T=0.00544/1440 (TOPMED), T=0.001597/8 (1000Genomes), T=0.005712/212 (ExAC) | Caucasian | [[28]](https://paperpile.com/c/GWUdu2/Ngwg) |

^&^ As reported in the original publication. NA: Not Available; Ref: Reference; NHW: Non-Hispanic whites.

References

[1. Guerreiro RJ, Lohmann E, Kinsella E, Brás JM, Luu N, Gurunlian N, et al. Exome sequencing reveals an unexpected genetic cause of disease: NOTCH3 mutation in a Turkish family with Alzheimer’s disease. Neurobiol Aging. 2012;33:1008.e17–23.](http://paperpile.com/b/GWUdu2/JFHH)

[2. Sassi C, Nalls MA, Ridge PG, Gibbs JR, Lupton MK, Troakes C, et al. Mendelian adult-onset leukodystrophy genes in Alzheimer’s disease: critical influence of CSF1R and NOTCH3. Neurobiol Aging. 2018;66:179.e17–179.e29.](http://paperpile.com/b/GWUdu2/n9sd)

[3. Patel D, Mez J, Vardarajan BN, Staley L, Chung J, Zhang X, et al. Association of Rare Coding Mutations With Alzheimer Disease and Other Dementias Among Adults of European Ancestry. JAMA Netw Open. 2019;2:e191350.](http://paperpile.com/b/GWUdu2/cf0c)

[4. Guerreiro R, Wojtas A, Bras J, Carrasquillo M, Rogaeva E, Majounie E, et al. TREM2 variants in Alzheimer’s disease. N Engl J Med. 2013;368:117–27.](http://paperpile.com/b/GWUdu2/CLlY)

[5. Jonsson T, Stefansson H, Steinberg S, Jonsdottir I, Jonsson PV, Snaedal J, et al. Variant of TREM2 associated with the risk of Alzheimer’s disease. N Engl J Med. 2013;368:107–16.](http://paperpile.com/b/GWUdu2/tpwt)

[6. Korvatska O, Leverenz JB, Jayadev S, McMillan P, Kurtz I, Guo X, et al. R47H Variant of TREM2 Associated With Alzheimer Disease in a Large Late-Onset Family: Clinical, Genetic, and Neuropathological Study. JAMA Neurol. 2015;72:920–7.](http://paperpile.com/b/GWUdu2/iQgO)

[7. Sims R, van der Lee SJ, Naj AC, Bellenguez C, Badarinarayan N, Jakobsdottir J, et al. Rare coding variants in PLCG2, ABI3, and TREM2 implicate microglial-mediated innate immunity in Alzheimer’s disease. Nat Genet. 2017;49:1373–84.](http://paperpile.com/b/GWUdu2/GJAL)

[8. De Roeck A, Van Broeckhoven C, Sleegers K. The role of ABCA7 in Alzheimer’s disease: evidence from genomics, transcriptomics and methylomics. Acta Neuropathol. 2019;138:201–20.](http://paperpile.com/b/GWUdu2/sYZV)

[9. De Roeck A, Van den Bossche T, van der Zee J, Verheijen J, De Coster W, Van Dongen J, et al. Deleterious ABCA7 mutations and transcript rescue mechanisms in early onset Alzheimer’s disease. Acta Neuropathol. 2017;134:475–87.](http://paperpile.com/b/GWUdu2/TEls)

[10. Pottier C, Hannequin D, Coutant S, Rovelet-Lecrux A, Wallon D, Rousseau S, et al. High frequency of potentially pathogenic SORL1 mutations in autosomal dominant early-onset Alzheimer disease. Mol Psychiatry. 2012;17:875–9.](http://paperpile.com/b/GWUdu2/YjVV)

[11. Nicolas G, Charbonnier C, Wallon D, Quenez O, Bellenguez C, Grenier-Boley B, et al. SORL1 rare variants: a major risk factor for familial early-onset Alzheimer’s disease. Mol Psychiatry. 2016;21:831–6.](http://paperpile.com/b/GWUdu2/CjNg)

[12. Verheijen J, Van den Bossche T, van der Zee J, Engelborghs S, Sanchez-Valle R, Lladó A, et al. A comprehensive study of the genetic impact of rare variants in SORL1 in European early-onset Alzheimer’s disease. Acta Neuropathol. 2016;132:213–24.](http://paperpile.com/b/GWUdu2/bqdd)

[13. Vardarajan BN, Zhang Y, Lee JH, Cheng R, Bohm C, Ghani M, et al. Coding mutations in SORL1 and Alzheimer disease. Ann Neurol. 2015;77:215–27.](http://paperpile.com/b/GWUdu2/fmCb)

[14. Cuccaro ML, Carney RM, Zhang Y, Bohm C, Kunkle BW, Vardarajan BN, et al. SORL1 mutations in early- and late-onset Alzheimer disease. Neurol Genet. 2016;2:e116.](http://paperpile.com/b/GWUdu2/1mjv)

[15. Thonberg H, Chiang H-H, Lilius L, Forsell C, Lindström A-K, Johansson C, et al. Identification and description of three families with familial Alzheimer disease that segregate variants in the SORL1 gene. Acta Neuropathol Commun. 2017;5:43.](http://paperpile.com/b/GWUdu2/DUTN)

[16. Le Guennec K, Tubeuf H, Hannequin D, Wallon D, Quenez O, Rousseau S, et al. Biallelic Loss of Function of SORL1 in an Early Onset Alzheimer’s Disease Patient. J Alzheimers Dis. 2018;62:821–31.](http://paperpile.com/b/GWUdu2/4opf)

[17. Vardarajan BN, Ghani M, Kahn A, Sheikh S, Sato C, Barral S, et al. Rare coding mutations identified by sequencing of Alzheimer disease genome-wide association studies loci. Ann Neurol. 2015;78:487–98.](http://paperpile.com/b/GWUdu2/099O)

[18. Tan M-S, Yu J-T, Jiang T, Zhu X-C, Guan H-S, Tan L. Genetic variation in BIN1 gene and Alzheimer’s disease risk in Han Chinese individuals. Neurobiol Aging. 2014;35:1781.e1–8.](http://paperpile.com/b/GWUdu2/YPnH)

[19. Bettens K, Brouwers N, Engelborghs S, Lambert J-C, Rogaeva E, Vandenberghe R, et al. Both common variations and rare non-synonymous substitutions and small insertion/deletions in CLU are associated with increased Alzheimer risk. Mol Neurodegener. 2012;7:3.](http://paperpile.com/b/GWUdu2/pPwZ)

[20. Naj AC, Leonenko G, Jian X, Grenier-Boley B, Dalmasso MC, Bellenguez C, et al. Genome-wide meta-analysis of late-onset Alzheimer’s disease using rare variant imputation in 65,602 subjects identifies novel rare variant locus NCK2: The International Genomics of Alzheimer’s Project (IGAP) [Internet]. bioRxiv. medRxiv; 2021. Available from:](http://paperpile.com/b/GWUdu2/3Rts) <http://medrxiv.org/lookup/doi/10.1101/2021.03.14.21253553>

[21. Schwartzentruber J, Cooper S, Liu JZ, Barrio-Hernandez I, Bello E, Kumasaka N, et al. Author Correction: Genome-wide meta-analysis, fine-mapping and integrative prioritization implicate new Alzheimer’s disease risk genes. Nat Genet. 2021;53:585–6.](http://paperpile.com/b/GWUdu2/Tv87)

[22. Logue MW, Schu M, Vardarajan BN, Farrell J, Bennett DA, Buxbaum JD, et al. Two rare AKAP9 variants are associated with Alzheimer’s disease in African Americans. Alzheimers Dement. 2014;10:609–18.e11.](http://paperpile.com/b/GWUdu2/Xsho)

[23. Vardarajan BN, Barral S, Jaworski J, Beecham GW, Blue E, Tosto G, et al. Whole genome sequencing of Caribbean Hispanic families with late-onset Alzheimer’s disease. Ann Clin Transl Neurol. 2018;5:406–17.](http://paperpile.com/b/GWUdu2/i4j6)

[24. Wetzel-Smith MK, Hunkapiller J, Bhangale TR, Srinivasan K, Maloney JA, Atwal JK, et al. A rare mutation in UNC5C predisposes to late-onset Alzheimer’s disease and increases neuronal cell death. Nat Med. 2014;20:1452–7.](http://paperpile.com/b/GWUdu2/ci0S)

[25. Jiao B, Liu X, Tang B, Hou L, Zhou L, Zhang F, et al. Investigation of TREM2, PLD3, and UNC5C variants in patients with Alzheimer’s disease from mainland China. Neurobiol Aging. 2014;35:2422.e9–2422.e11.](http://paperpile.com/b/GWUdu2/vM72)

[26. Cukier HN, Kunkle BK, Hamilton KL, Rolati S, Kohli MA, Whitehead PL, et al. Exome Sequencing of Extended Families with Alzheimer’s Disease Identifies Novel Genes Implicated in Cell Immunity and Neuronal Function. J Alzheimers Dis Parkinsonism [Internet]. 2017;7. Available from:](http://paperpile.com/b/GWUdu2/QLN2) <http://dx.doi.org/10.4172/2161-0460.1000355>

[27. Dalmasso MC, Brusco LI, Olivar N, Muchnik C, Hanses C, Milz E, et al. Transethnic meta-analysis of rare coding variants in PLCG2, ABI3, and TREM2 supports their general contribution to Alzheimer’s disease. Transl Psychiatry. 2019;9:55.](http://paperpile.com/b/GWUdu2/QzhR)

[28. Conway OJ, Carrasquillo MM, Wang X, Bredenberg JM, Reddy JS, Strickland SL, et al. ABI3 and PLCG2 missense variants as risk factors for neurodegenerative diseases in Caucasians and African Americans. Mol Neurodegener. 2018;13:53.](http://paperpile.com/b/GWUdu2/Ngwg)
